# Individual and Environmental Impacts on Sexual Health of Caribbean youth

**DOI:** 10.1100/tsw.2006.150

**Published:** 2006-06-22

**Authors:** Sarah J. Lerand, Marjorie Ireland, Robert W. Blum

**Affiliations:** ^1^ Department of Pediatrics, Center for Adolescent Health and Development at the University of Minnesota, Minneapolis, MN, USA; ^2^ Bloomberg School of Public Health, Department of Population and Family Health Services in Baltimore, MD, USA

**Keywords:** risk and protective factors, sexual activity, Caribbean, mental health, rage, abuse, connectedness, United States

## Abstract

Individual health risk behaviors among Caribbean youth account for the majority of adolescent morbidity and mortality in that area. This study explores the associations between individual factors, socioenvironmental factors, and sexual health—related behaviors in Caribbean youth. Data from the 1995 Caribbean Youth Health Survey, a nine-country, cross-sectional study completed by 15,695 in-school youth 10—18 years of age were analyzed. One-third of the sample (n = 5,060) reporting sexual activity was analyzed. This study examined age at first sexual intercourse, number of sexual partners, history of pregnancy, and condom use. The predictor variables were rage, depressed mood, expectation of early death, self-reported school performance, parental mental health or substance abuse problems, and family connectedness. Bi- and multivariate analyses were done separately for males and females, controlling for age, to examine associations between individual and socioenvironmental factors and sexual health behaviors. In the multivariate model, there were associations between rage, abuse, family mental health and substance use, anticipation of early death, and many of the outcome variables in males and females. Family connectedness and positive self-reported school status were correlated with greater condom use at last intercourse in males. Family connectedness was correlated with older age at first sexual intercourse. Depressed mood was not correlated with any of the outcome variables.The findings of the study demonstrate an association between individual and socioenvironmental factors and sexual health behaviors in the lives of Caribbean youth. Strong associations between rage and physical/sexual abuse and risky sexual behaviors are of notable concern.

## INTRODUCTION

In several Caribbean countries, the Human Immunodeficiency Virus (HIV) prevalence rates are approaching levels seen only in Sub-Saharan Africa. As in other countries, now the majority of new cases of HIV are from heterosexual transmission. Over one-half of the new infections in Caribbean countries occur in youths aged 15–24, and women now account for half of all new cases of HIV[1,2].

The first goal of this study was to examine the frequency of behaviors known to increase the risk of acquiring a sexually transmitted infection (STI) or HIV in Caribbean youth. Sexual behaviors and/or outcomes that have been associated with higher rates of STI and HIV transmission include early age of sexual intercourse, multiple sexual partners, not using a condom at last intercourse, and pregnancy[3,4,5]. Among adolescents in Jamaica with an STI, 7.6% of them are coinfected with HIV[6].

The second goal was to compare individual and socioenvironmental factors of sexually active adolescent females and males who engage in high-risk sexual behaviors compared with those who had lower-risk sexual activity. Blum et al.[18] have shown that risk and protective factors in the lives of Caribbean youth are similar to that of U.S. youth. In this present analysis, we will apply the risk and resiliency framework to high-risk sexual behavior in Caribbean youth. We hypothesize that risk and protective factors will be different in males compared to females. We expect that environmental factors will influence males more due to the male "machismo" culture in the Caribbean[7].

## Literature Review

Rates of sexual activity in Caribbean youth range from 25–50% of school-going adolescents[8,9,10]. Many sexually active Caribbean adolescents engage in sexual behaviors associated with HIV transmission[2]. It is estimated that between 20 and 66% of sexually active youth report sexual activity before age 13[9,10,11,12]. Halcon et al.[9] reported that males were three times more likely to have multiple sexual partners than females. The prevalence of condom use in the Caribbean is low[3,4,5]. In a study of Jamaican youth, over 40% of sexually active males had not used contraception at last intercourse[6]. In a cohort of Jamaican youth that were seen at an STI clinic, only 4% had consistent condom use[13]. Approximately 10% of school-going adolescents had been pregnant or had gotten someone pregnant[8,9]. The majority of these pregnancies had not been planned[8].

A recent study of adolescents in Tobago found that reported rates of depressive symptoms were similar to those in developed countries[14]. Depression has been linked with higher STI acquisition, including HIV, and pregnancy[11,15]. One study of high-risk youth demonstrated that teenage women who had early intercourse, did not use contraception, and had been pregnant were more likely to be depressed, feel a failure, have low self-esteem, and little sense of control over their lives. Males showed similar associations with depression and ineffective contraception and pregnancy, but not in the other areas[16]. Of additional concern is that depression has been associated with unprotected sex in HIV-infected adolescents due to more apathy and less concern about exposure[17].

Rage has also been correlated with increased risk-taking behaviors. A survey of Caribbean youth showed that two out of five report they sometimes, or most of the time, think about hurting or killing someone else. An additional 4.9% reported that they almost always think about hurting or killing others[9]. Blum et al.[18] reported that rage was associated with early sexual debut in Caribbean youth. Feelings of rage or aggression have been correlated with other risk-taking behaviors in adolescents such as use of alcohol and other drugs, sexual intercourse, problems with peer and family relations, and reporting more mental health symptoms[19] and school problems[20,21].

Adolescents who feel vulnerable to harm or early death often engage in riskier behaviors[22,23]. Death, AIDS, and neighborhood violence worry Caribbean youth[9]. Higher HIV/AIDS vulnerability predicted higher HIV risk behavior in adolescents. A risk, such as acquiring HIV, may be less important to an adolescent who already feels that they will die at an early age[22].

Rates of sexual and physical abuse in the Caribbean are high[6]. Of the adolescents who have experienced intercourse, between 10 and 50% report that their first intercourse was forced[9,10]. Adolescents who have been physically or sexually abused show higher levels of depression, suicidal ideation, anxiety, and hopelessness than their nonabused peers[24,25,26,27]. In addition, a number of studies have shown that exposure to childhood physical and sexual abuse increases risk-taking behaviors in adolescents[11,26,27,28,29].

Adolescents who feel vulnerable to harm or hopelessness in their home and community environment have been shown to increase risk behaviors. In the Caribbean, Blum et al. found that knowing a friend or family member who has attempted or completed suicide reported more health risk behaviors. Of the adolescents surveyed, 20% reported that they knew someone who had tried to kill themselves.

Family and school connectedness has been shown to be protective in the lives of Caribbean youth[18]. The role of family and parental love is important in the lives of Caribbean adolescents. Parental love and family connectedness have been correlated with less sexual activity[7,12,18]. Youth who postpone sexual activity report greater support, supervision, and involvement with parents. Adolescents who remain in school engage in less risk-taking behaviors[30]; however, school drop-out rates in many countries remain high[31].

## METHODS

The present study was a secondary analysis of data from a nine-country, cross-sectional study of adolescent health in the Caribbean completed by 15,695 young people 10–18 years of age.

### Instrument Development

The survey instrument was developed by an international group who met in 1995 to review previously reviewed surveys of adolescent risk and protective factors. Subsequently, Maternal and Child Health representatives from 19 Caribbean nations reviewed the initial survey instrument. The survey was pilot tested in Barbados, St. Lucia, and Antigua (n = 105). The decision to use the core survey instrument in all the countries decreased cost and also helped to ensure comparable results across countries. Country-specific questions could be added to the end of the core survey to address unique issues of individual countries[9,18].

### Sampling

Of the 19 CARICOM countries, nine submitted data for analysis. The participating countries included the English-speaking countries of Antigua, Bahamas, Barbados, British Virgin Islands, Dominica, Grenada, Guyana, Jamaica, and St. Lucia. The sampling strategy used an in-school, classroom frame. The sample drawn in each country was of significant size to allow for a power of 0.80 to detect differences between genders and among age groups within each country, as well as between country differences. An oversampling of 20% was done to account for absentees on the day of survey administration. Representatives from the Ministries of Health in each country determined the sampling procedures and took responsibility for training data survey administrators.

The survey consisted of 87 multiple-choice questions addressing school performance; school environment; tobacco, alcohol and other drug use; sexual and reproductive health; physical and sexual abuse; moral behavior; violence; mental health and suicide; family characteristics; religiosity; general health; health care; and nutrition. Passive parental consent was obtained, which met the standards for the community. The Institutional Review Board of the Human Subjects Review Committee of the University of Minnesota approved the original study and present secondary analysis. The study design and data collection methods are detailed elsewhere[9,18].

In the present analyses, the sample included only those youth who responded that they had been sexually active (n = 5,060, 32% of those surveyed). Of the sexually active adolescents, 60% were male and 40% were female; 10.8% were ≤13 years, 44.8% were 13–15 years old, and 44.4% were 16 years or older.

Four outcome variables were used for the present analysis, each of which was dichotomized: (1) early sexual debut was defined as having had intercourse at or below the age of 13; (2) multiple sexual partners was defined as having four or more partners over one's lifetime; (3) pregnancy/caused pregnancy was defined as having one or more of these events having occurred; (4) condom use was defined as no condom used at last sexual intercourse.

The risk and resiliency framework has been used to explore individual and environmental factors that influence adolescent behavior. It has proven useful in identifying adolescents who are vulnerable to harm and those factors that may protect them from potential negative outcomes[32,33]. Blum et al.[18] have shown this model also applied to Caribbean youth. This paper uses this framework to explore associations between individual factors, socioenvironmental factors, and sexual health–related behaviors (see Fig. 1).

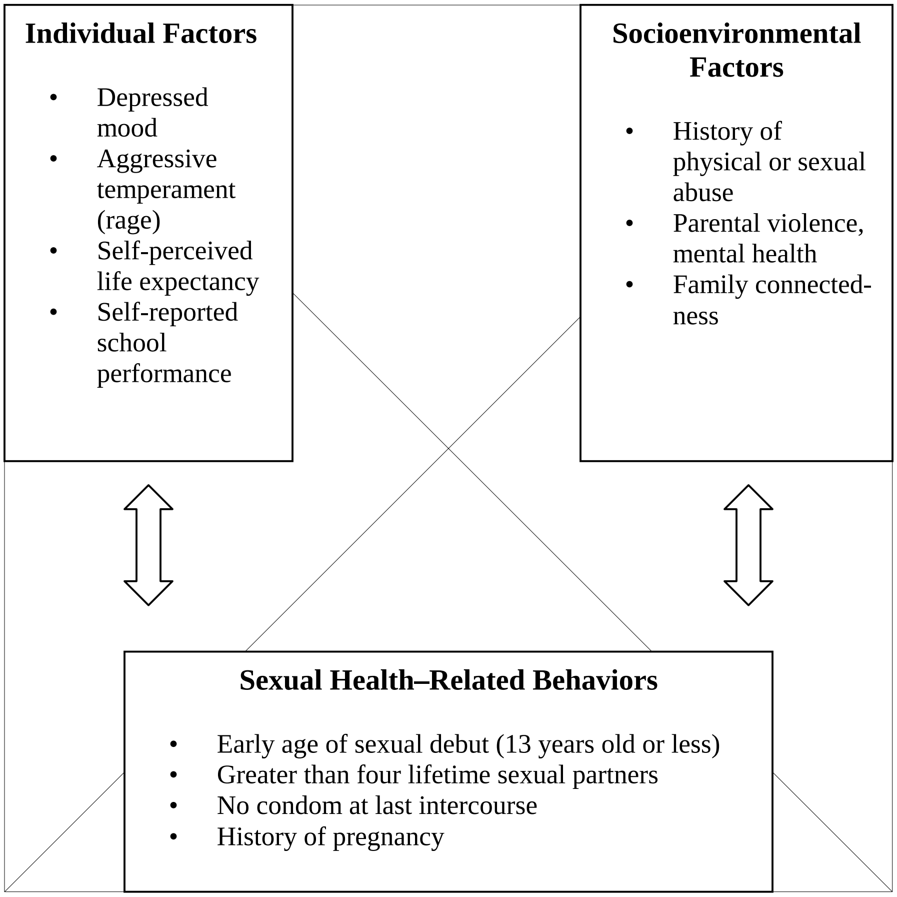

**FIGURE 1.** Interaction between individual and socioenvironmental factors and sexual health–related behaviors in Caribbean youth.

Seven dichotomous variables examining individual and environmental risk and protective factors were included in this study. The individual factors included: rage, thoughts about wanting to hurt or kill someone some or all of the time (single variable); depressed mood, an affirmative response to either of two questions exploring one's sense of self as sad or irritable much or all or the time, and/or feeling so down or discouraged that nothing is worthwhile[34]; expectation of early death, thinking they will not live to be 25 years old (single variable); school performance, self-report of average or above average performance vs. below average performance (single variable).

The environmental factors examined included: abuse, ever having been physically or sexually abused (single variable); family environment**,** a positive endorsement to any of five items related to parental substance use and mental health (parental drinking, drug use, mental health, suicide attempts, and violence); family connectedness, a six-item scale based on responses to questions about feeling cared for by parents, being able to tell parents their problems, and being paid attention to or understood by the family (Chronbach alpha = 0.76).

## Statistical Analysis

Descriptive univariate analyses were performed to examine the frequency distribution of the outcome variables. We performed logistic regressions for each pair of dependent and independent variables, including age in the logistic model. Consequently, the statistic that we report is the odds ratio, as well as the correlated 95% confidence interval, and the $p$ value. In addition, we performed fully multivariate logistic regression to evaluate the relative importance of all the independent variables, controlling for all others. We felt that since age is such an important confounder for sexual issues, it was desirable to control for age in the multivariate analyses.

## RESULTS

Results for males and females were reported separately because of the differences between the two groups. Table 1 details the sexual behavior of the Caribbean adolescents sampled. Table 2 summarizes results of the bivariate analyses and Table 3, the multivariate analysis by gender.

**TABLE 1**
**Frequencies of Sexual Behaviors in Caribbean Adolescents**

| Sexually Active Adolescents (n = 5,060) | Female % | Male % |
|---|---|---|
| Age at first intercourse | | |
| 10–11 | 21.6 | 38.9 |
| 12–13 | 33.1 | 30.6 |
| 14–15 | 29.3 | 21.7 |
| ≥16 | 16.0 | 8.8 |
| Total number of sexual partners | | |
| 1 | 49.0 | 23.7 |
| 2 | 20.0 | 15.6 |
| 3 | 10.9 | 15.2 |
| 4 | 5.4 | 9.9 |
| 5+ | 14.7 | 35.6 |
| Has been or has gotten someone pregnant | 10.4 | 15.0 |
| Used condom during most recent intercourse | 62.9 | 52.1 |

**Males — Individual Factors***:* Three individual factors were significantly correlated with high-risk sexual behaviors among adolescent males. Feelings of rage were correlated with early sexual debut (OR = 1.62, $p < 0.001$), four or more lifetime sexual partners (OR = 1.24, $p = 0.01$), and history of getting someone

**TABLE 2**
**Bivariate Analysis of Risk and Protective Factors for High-Risk Sexual Behaviors in Caribbean Youth Reporting Sexual Activity (n = 5,060)**

| | Early Sexual Debut[a] | No Condom | Multiple Partners | Pregnancy |
|---|---|---|---|---|
| | OR (95% CI) *p* value | | | |
| **Risk factors:** | | | | |
| Rage | | | | |
| Boys | 1.73 (1.53–1.97) *** | 1.06 (0.90–1.25) | 1.29 (1.10–1.50) ** | 1.42 (1.13–1.79) ** |
| Girls | 1.99 (1.71–2.33) *** | 0.97 (0.74–1.13) | 1.79 (1.39–2.32) *** | 1.24 (0.90–1.71) |
| Depressed mood | | | | |
| Boys | 1.22 (1.01–1.64) * | 1.51 (1.16–1.96) ** | 0.90 (0.70–1.16) | 1.03 (0.72–1.47) |
| Girls | 1.48 (1.20–1.82) ** | 1.22 (0.94–1.58) | 1.01 (0.76–1.36) | 1.61 (1.12–2.32 ) * |
| Early death | | | | |
| Boys | 1.22 (1.01–1.49) * | 1.35 (1.04–1.76) * | 1.16 (0.91–1.48) | 2.50 (1.86–3.36) *** |
| Girls | 1.36 (1.09–1.70) ** | 1.33 (0.97–1.83) | 1.29 (0.91–1.83) | 1.89 (1.27–2.81) ** |
| Abuse | | | | |
| Boys | 1.64 (1.38–1.95) *** | 1.54 (1.26–1.88) *** | 1.05 (0.87–1.27) | 2.41 (1.88–3.08) *** |
| Girls | 2.67 (2.25–3.17) *** | 1.64 (1.31–2.04) *** | 1.54 (1.21–1.97) ** | 2.17 (1.57–3.00) *** |
| Family problems | | | | |
| Boys | 1.76 (1.50–2.05) *** | 1.54 (1.27–1.85) *** | 1.41 (1.18–1.68) *** | 1.65 (1.30–2.10) *** |
| Girls | 1.42 (1.19–1.69) *** | 1.23 (0.98–1.54) | 1.32 (1.03–1.70) * | 1.32 (0.94–1.84) |
| **Protective factors:** | | | | |
| School performance | | | | |
| Boys | 0.77 (0.62–0.96) * | 0.63 (0.46–0.85) ** | 1.20 (0.90–1.61) | 0.78 (0.51–1.17) |
| Girls | 0.63 (0.48–0.82) ** | 0.92 (0.63–1.36) | 0.84 (0.54–1.31) | 0.65 (0.36-1.18) |
| Family connectedness | | | | |
| Boys | 0.57 (0.45–0.73) *** | 0.43 (0.31–0.59) *** | 0.95 (0.71–1.28) | 0.54 (0.36–0.82) ** |
| Girls | 0.30 (0.22–0.39) *** | 0.66 (0.45–0.97) * | 0.73 (0.47–1.13) | 0.47 (0.26–0.83) ** |

Legend: [a]Reference group is all adolescents who have not had intercourse by age 14 (n = 6,369). * = $p \leq 0.05$; ** = $p < 0.01$; *** = $p \leq 0.0001$.

pregnant (OR = 1.33, $p = 0.035$). Males who did not think that they would live to age 25 had a two and a half times greater odds risk of getting someone pregnant (OR = 2.53, $p < 0.001$). Males who reported doing better in school were at a lower odds risk of not using a condom at last intercourse (OR = 0.63, $p =$ < 0.001). Depressed mood was not correlated with any of the outcome variables.

**Males — Environmental Factors**: A history of stressful life events had an increased odds risk of involvement in high-risk sexual behaviors. Specifically, a history of physical or sexual abuse had a greater odds risk of getting someone pregnant (OR = 2.39, $p$ <0.001), early sexual debut (OR = 1.52, $p <$ 0.001), and not using condoms at last intercourse (OR = 1.45, $p < 0.001$). Males who reported a history of family violence, mental health, or substance use problems had a one and a half times odds risk of early sexual debut, not using a condom at last intercourse, or greater than four lifetime sexual partners (OR = 1.64, $p$ <0.001; OR = 1.40, $p = 0.002$; OR = 1.40, $p < 0.001$). Males who felt connected to their families had a lesser odds risk of not using a condom at last intercourse (OR = 0.46 $p$ <0.001).

**TABLE 3**
**Multivariate Analysis of Risk and Protective Factors for High-Risk Sexual Behaviors in Caribbean Youth Reporting Sexual Activity, Controlled for Age (n = 5,060)**

| | Early Sexual Debut[a] | No Condom | Multiple Partners | Pregnancy |
|---|---|---|---|---|
| | OR (95% CI) *p* value | | | |
| **Risk factors:** | | | | |
| Rage | | | | |
| Boys | 1.62 (1.41-1.88) *** | 1.02 (0.85-1.23) | 1.24 (1.05-1.48) * | 1.33 (1.02-1.74) * |
| Girls | 1.78 (1.48-2.13) *** | 0.78 (0.61-0.99) * | 1.95 (1.45-2.62) *** | 1.19 (0.81-1.76) |
| Depressed mood | | | | |
| Boys | 1.01 (0.77-1.33) | 1.26 (0.94-1.70) | 0.83 (0.62-1.10) | 0.69 (0.46-1.05) |
| Girls | 0.82 (0.64-1.06) | 1.11 (0.82-1.51) | 0.72 (0.51-1.03) | 1.10 (0.71-1.74) |
| Early death | | | | |
| Boys | 1.05 (0.84-1.32) | 1.04 (0.77-1.40) | 1.14 (0.87-1.51) | 2.53 (1.80-3.57) *** |
| Girls | 1.28 (0.99-1.65) | 1.16 (0.82-1.66) | 1.31 (0.89-1.93) | 1.87 (1.19-2.92) ** |
| Abuse | | | | |
| Boys | 1.52 (1.26-1.84) *** | 1.45 (1.16-1.81) ** | 1.04 (0.84-1.27) | 2.39 (1.82-3.13) *** |
| Girls | 2.40 (1.99-2.90) *** | 1.70 (1.30-2.11) *** | 1.57 (1.20-2.05) ** | 1.89 (1.30-2.74) ** |
| Family problems | | | | |
| Boys | 1.64 (1.37-1.95) *** | 1.40 (1.13-1.72) ** | 1.40 (1.15- 1.70) ** | 1.25 (0.95-1.65) |
| Girls | 1.02 (0.83-1.26) | 1.17 (0.90-1.52) | 1.10 (0.83-1.47) | 0.96 (0.64-1.44) |
| **Protective factors:** | | | | |
| School performance | | | | |
| Boys | 0.82 (0.64-1.05) | 0.63 (0.45-0.89) ** | 1.18 (0.86-1.61) | 0.80 (0.50-1.25) |
| Girls | 0.74 (0.55-1.01) | 0.96 (0.63-1.48) | 0.92 (0.56-1.51) | 0.91 (0.47-1.76) |
| Family connectedness | | | | |
| Boys | 0.78 (0.58-1.04) | 0.46 (0.32-0.66) *** | 1.08 (0.77-1.52) | 0.99 (0.60-1.63) |
| Girls | 0.57 (0.40-0.80) ** | 0.83 (0.53-1.31) | 1.07 (0.64-1.80) | 0.68 (0.34-1.37) |

Legend: [a]Reference group is all adolescents who have not had intercourse by age 14 (n = 6,369). * = $p \leq 0.05$; ** = $p < 0.01$; *** = $p \leq 0.0001$.

**Females — Individual Factors**: Two individual factors were correlated with high-risk sexual behaviors among adolescent females. Consistent with males, females who reported rage had an increased odds risk of early sexual debut and multiple sexual partners (OR = 1.78, $p$ <0.001; OR = 1.95, $p$ <0.001). Females who expressed feelings of rage had a lower odds risk of not using a condom at last intercourse (OR = 0.78, $p$ = 0.043); rage was not correlated with condom use in males. On the other hand, females who believed that they would not live to age 25 had a one and a half times greater odds risk of reporting a history of pregnancy (OR = 1.87, $p$ < 0.001). Neither depressed mood nor self-reported school performance were correlated with high-risk sexual behaviors.

**Females — Environmental Factors**: A history of sexual or physical abuse was correlated with a higher likelihood of early age at first sexual intercourse, four or more lifetime sexual partners, and a history of pregnancy (OR = 2.40, $p$ < 0.001; OR = 1.57, $p$ = 0.001; OR = 1.89, $p$ < 0.001). In contrast to males, a history of family violence, mental health, or substance abuse problems were not correlated with higher reported sexual behaviors for females. Females who felt connected to their family had a one and a half times decreased odds risk of reporting early sexual debut when compared with less connected peers (OR = 0.57, $p$ = 0.001).

## DISCUSSION

The present study examined risk and protective factors for high-risk sexual behaviors in Caribbean adolescents. The examination of these risk and protective factors in the lives of Caribbean youth is important for the development of STI and HIV prevention programs. Since this study was only in school-attending youth and inconsistency in reporting of sexual activity has been reported previously[35], the estimates of frequencies of high-risk sexual behaviors are probably an overestimate. As hypothesized, there were differences among risk and protective factors in males and females. Overall, females were less likely to engage in high-risk sexual behaviors, and this is consistent with previous studies[36,37].

Risk and protective factors for high-risk sexual behaviors in this analysis were similar for males and females. For males, rage, a history of abuse, mental illness, and/or substance abuse in the family were associated with three out of the four high-risk sexual behaviors examined. The belief that they may have an early death was only associated with having caused a pregnancy. Further research is needed to examine the social and psychological impacts of adolescent male fatherhood in the Caribbean. Connectedness to family and school were associated with a higher odds of using a condom at last sexual intercourse, but were not found to be protective for any of the other sexual behaviors examined. One explanation for this is that males who feel that their family and peers approve of condom use may be more likely to use them.

A history of abuse was a risk factor for all four of the sexual behaviors examined in this study. Rates of abuse in males and females in the Caribbean are high. In other studies, a history of abuse has been associated with risk-taking behaviors in adolescents[26,29]. However, in the case of sexual abuse, females are not able to avoid situations that increase risk of STIs and HIV. Rage was a risk factor for females having early age of sexual debut and multiple sexual partners. However, it was protective for condom use at last intercourse. One hypothesis is that females who feel rage may have more self-efficacy surrounding condom use than other females. The belief that they may have an early death was only associated with having gotten pregnant. It is well documented that adolescent childbearing is associated with significant social and economic risks in the Caribbean. Females who felt connected to their families were more likely to be older at age of first intercourse than those who did not feel connected to their families. However, it was not protective for the other three sexual behaviors examined. Possibly, once an adolescent decides to engage in sexual intercourse, connectedness to the family may not be as important in influencing their decisions.

There are several factors that limit the generalizability of these findings. First, because of the cross-sectional design, causality cannot be determined. In addition, this study did not compare adolescents who were sexually active with those who were not. Risk factors in the sexually active population may be different from those who are not sexually active. This study included only adolescents attending school. In some Caribbean countries, less than half of older adolescents attend secondary school and this number is higher for disadvantaged populations[31]. In addition, students who were absent from school are more likely to be involved in risky health behaviors[38]. The present analyses rely on self-report data. Eggleston et al.[35] examined the consistency of self-reports of sexual activity among young adolescents in Jamaica and found consistency to be a problem, especially among males. The abuse variable consisted of both physical and sexual abuse. Therefore, we were not able to determine if the reason for early first sexual intercourse was because they had been sexually abused. The depression variable only included one question that asked about overall mood, and was not validated. This may explain why depression was not a significant risk factor for any of the sexual behaviors, as has been found in other studies. Finally, the data are now 10 years old. However, shifts in personal and environmental factors and how they influence sexual behaviors are still valid. The large sample size does increase the validity of these findings.

## CONCLUSIONS

The findings in this study demonstrated associations between individual and socioenvironmental factors and sexual health behaviors in the lives of Caribbean youth. One area to explore further is the role of the family and the lives of Caribbean youth. The variables that had the strongest associations with the sexual behaviors in this sample of youth were history of physical or sexual abuse, rage, family history of mental illness and substance abuse, and family connectedness. Potentially, the family environment in which the adolescent lives could influence all of these factors.

Of notable concern from these findings were the strong associations between rage and sexual behaviors in Caribbean adolescents. Questions about rage have not been routinely asked in other surveys of adolescent behavior. This variable and the factors that may contribute to an adolescent's feeling of rage must be further examined and validated. Once this is done, programs to address this problem can be developed.

As policy and programs are being developed to reduce teen pregnancy, STIs, and HIV risk, these individual and environmental factors need to be taken into consideration. These factors will be useful when developing programs that identify potential risk factors and work to enhance adolescent development.

## ACKNOWLEDGMENTS

This manuscript was developed while the author, Sarah J. Lerand, was a fellow in training in the Leadership Education in Adolescent Health Program at the University of Minnesota's Center for Adolescent Health and Development. It was supported in part from grant 1-T71-MC00025-01 of the Maternal and Child Health Bureau.

## BIOSKETCHES

**Sarah J. Lerand, MD**, Center for Adolescent Health and Development, Department of Pediatrics, University of Minnesota, 200 Oak Street, SE, Suite 260, Minneapolis, MN 55455. E-mail: lera0005@umn.edu

**Marjorie Ireland, PhD**, Center for Adolescent Health and Development, Department of Pediatrics, University of Minnesota, 200 Oak Street, SE, Suite 260, Minneapolis, MN 55455. E-mail: irela002@umn.edu

**Robert W. Blum, MD, MPH, PhD,** William H. Gates Senior Professor Chair at Bloomberg School of Public Health, Department of Population and Family Health Services in Baltimore, MD. E-mail: rblum@jhsph.edu
